# A Revolution toward Gene-Editing Technology and Its Application to Crop Improvement

**DOI:** 10.3390/ijms21165665

**Published:** 2020-08-07

**Authors:** Sunny Ahmar, Sumbul Saeed, Muhammad Hafeez Ullah Khan, Shahid Ullah Khan, Freddy Mora-Poblete, Muhammad Kamran, Aroosha Faheem, Ambreen Maqsood, Muhammad Rauf, Saba Saleem, Woo-Jong Hong, Ki-Hong Jung

**Affiliations:** 1 College of Plant Sciences and Technology Huazhong Agricultural University, Wuhan 430070, China; sunny.ahmar@yahoo.com (S.A.); sumbulsaeed717@gmail.com (S.S.); hafeezbiotech@webmail.hzau.edu.cn (M.H.U.K.); shahidbiochem@webmail.hzau.edu.cn (S.U.K.); 2 Institute of Biological Sciences, University of Talca, 2 Norte 685, Talca 3460000, Chile; morapoblete@gmail.com; 3 Key Laboratory of Arable Land Conservation (Middle and Lower Reaches of Yangtze River), Ministry of Agriculture, College of Resources and Environment, Huazhong Agricultural University, Wuhan 430070, China; kamiagrarian763@gmail.com; 4 Sate Key Laboratory of Agricultural Microbiology and State Key Laboratory of Microbial Biosensor, College of Life Sciences Huazhong Agriculture University Wuhan, Wuhan 430070, China; arushafaheem@hotmail.com; 5 Department of Plant Pathology, University College of Agriculture and Environmental Sciences, The Islamia University of Bahawalpur, Bahawalpur 63100, Pakistan; ambreenagrarian@gmail.com; 6 National Institute for Biotechnology and Genetic Engineering (NIBGE), Faisalabad 38000, Pakistan; rauf216@gmail.com; 7 Department of Bioscience, COMSATS Institute of Information Technology, Islamabad 45550, Pakistan; sabas.iiui@gmail.com; 8 Graduate School of Biotechnology & Crop Biotech Institute, Kyung Hee University, Yongin 17104, Korea; hwj0602@khu.ac.kr

**Keywords:** genome editing, CRISPR/Cas9, CRISPR/Cpf1, TALEN, crop improvement, speed editing strategy

## Abstract

Genome editing is a relevant, versatile, and preferred tool for crop improvement, as well as for functional genomics. In this review, we summarize the advances in gene-editing techniques, such as zinc-finger nucleases (ZFNs), transcription activator-like (TAL) effector nucleases (TALENs), and clustered regularly interspaced short palindromic repeats (CRISPR) associated with the Cas9 and Cpf1 proteins. These tools support great opportunities for the future development of plant science and rapid remodeling of crops. Furthermore, we discuss the brief history of each tool and provide their comparison and different applications. Among the various genome-editing tools, CRISPR has become the most popular; hence, it is discussed in the greatest detail. CRISPR has helped clarify the genomic structure and its role in plants: For example, the transcriptional control of Cas9 and Cpf1, genetic locus monitoring, the mechanism and control of promoter activity, and the alteration and detection of epigenetic behavior between single-nucleotide polymorphisms (SNPs) investigated based on genetic traits and related genome-wide studies. The present review describes how CRISPR/Cas9 systems can play a valuable role in the characterization of the genomic rearrangement and plant gene functions, as well as the improvement of the important traits of field crops with the greatest precision. In addition, the speed editing strategy of gene-family members was introduced to accelerate the applications of gene-editing systems to crop improvement. For this, the CRISPR technology has a valuable advantage that particularly holds the scientist’s mind, as it allows genome editing in multiple biological systems.

## 1. Introduction

The rapidly growing population and a wide range of competitive dairy products and meat are pushing agricultural output and expanding the demand for feed, food, biofuels, and livestock [1]. By 2050, the worldwide population will expand up to >9 billion, which may boost crop production demand by 100–110%. Consequently, the effective production of staple crops, such as *Oryza sativa* (rice), *Triticum aestivum* (wheat), *Zea mays* (maize), and *Glycine max* (soybean), will increase by just 38–67% [1,2]. Currently, numerous genome-editing tools and techniques have been adopted to overcome the problems arising in plants to compensate for the increased demand for food in the future [3]. Gene-editing techniques, such as engineered endonucleases/meganucleases (EMNs), zinc-finger nucleases (ZFNs), TAL effector nucleases (TALENs), and clustered regularly interspaced short palindromic repeats (CRISPR) [4], are important tools in plant research, as they allow the remodeling of future crops.

ZFNs were the first truly targeting protein reagents to revolutionize the genome manipulation area of research. ZFNs are binding domains for DNA that recognize three base pairs at the target site [5]. ZFNs have been commonly used for targeted genome modification in different plant species, such as *Arabidopsis thaliana* (*Arabidopsis*), *Nicotiana tabacum* (tobacco), and maize [6,7,8]. Another site-driven mutagenesis genome-editing system, TALENs, was defined first in plant pathogenic bacteria (*Xanthomonas*) and is based on a concept similar to that of ZFNs. TALENs target one nucleotide at the target site (instead of three), thus rendering TALENs precise [9]. TALENs were successfully used for genome editing in angiosperms and bryophytes [10,11].

**Figure 1:**
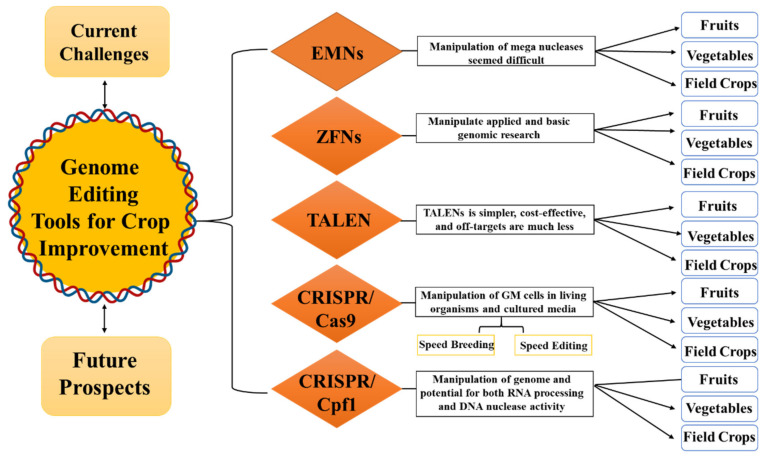
Overview of the different aspects covered in the present review related to genome editing in plants, such as its applications, challenges, and advantages.

Extensive investigation in this field led to the development of new genome-editing tools, such as CRISPR/Cas9 and CRISPR/Cpf1 [12,13]. Initially, these techniques were developed in prokaryotes, because there were no efficient genome-editing techniques for eukaryotes at specific sites. However, at the advent of eukaryotic genome editing, the CRISPR technology has revolutionized our ability to generate specific changes in crops [14]. The CRISPR system requires only the guide RNA sequence to be changed for each DNA target site. Under different circumstances, the usage and modification of CRISPR technology are quite simple and efficient [15,16]. In this review, we highlight the use of genome-editing techniques to achieve highly precise and desired modifications in plants, as well as examples of the application of EMNs, ZFNs, TALENs, and CRISPR/Cas9/Cpf1 in various plants (Figure 1).

## 2. The Journey from Engineered Meganucleases (EMNs) to CRISPR

### 2.1. Meganucleases (MNs)

Among endonucleases, meganucleases are characterized by the presence of a broad recognition site of about 12–40 bp. Because of their specific nature and long recognition site, these enzymes are regarded as the most precise restriction enzymes [17]. Therefore, meganucleases are also known as homing endonucleases. Repair in double-stranded breaks (DSBs) occurs via nonhomologous end joining (NHEJ), which is functionally responsible for knocking out genes in tobacco and *Arabidopsis* plants [18,19]. However, it is difficult to remodify meganucleases together with other genome-targeting techniques, because DNA-binding domains are often intermingled with the catalytic domain of meganucleases and cannot be detached from one another [20]. The outcomes of previous research demonstrated gene editing in plants by incorporating modified meganucleases in *Arabidopsis*, *Gossypium hirsutum* (cotton), and maize. However, additional efforts are needed to improve this approach, as the manipulation of meganucleases seems to be difficult. Hence, researchers focused on other more efficient, accurate, and simpler methods of gene editing, such as ZFNs, TALENs, and CRISPR.

### 2.2. Zinc-Finger Nucleases (ZFNs)

**Figure 2:**
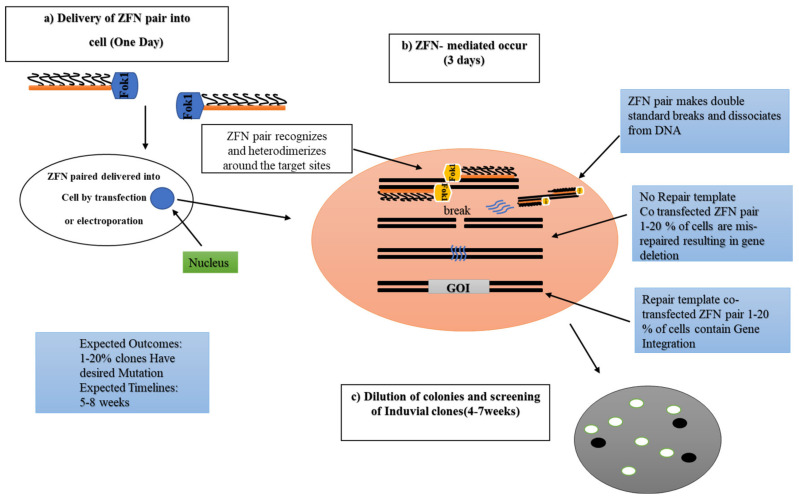
Processes of Zinc-finger nucleases (ZFNs). (**a**) Delivery of ZFNs into cells by transfection or electroporation. (**b**) ZFNs are fusions of the nonspecific DNA cleavage domain of the FokI restriction nuclease with zinc-finger proteins. ZFN dimers induce targeted and double-stranded breaks (DSBs) to stimulate the DNA damage response pathways. The binding specificity of the designed zinc-finger domain directs the ZFN to a particular genomic site. The boxes with blue color represent the mechanism of the ZFN genome-editing technique. (**c**) Cloning of ZFN-genome-edited cells and screening of positive clones by RT-PCR and sequencing analyses. This figure briefly modified from the source (https://www.sigmaaldrich.com/technical-documents/articles/life-science-innovations/targeted-genome-editing.html).

Zinc-finger nucleases (ZFNs) are one of the most efficient and effective tools for genome editing by targeting DSBs [21]. The first generation of genome-editing techniques based on ZFNs was developed using chimerically engineered nucleases. This approach was enabled by the discovery of the functional Cys2-His2 zinc-finger domain [4,22]. Fundamentally, the structural composition of ZFNs involves two domains: (1) The DNA-binding domain, which consists of 300–600 zinc-finger repeats [23]. Each zinc-finger repeat can monitor and read between 9 and 18 base pairs (bp); and (2) the DNA cleavage domain, which is known as the nonspecific cleavage domain of the type II restriction endonuclease Fok1 and acts as the DNA cleavage domain in ZFNs [24]. ZFNs contain two monomers attributed to their respective target sequences reversely flanking in between 5 and 6 bp of the DNA target [24,25]. Dimers containing Fok1 domains slice DNA within its flanking sequence (Figure 2). The specific sequence of 24–30 bp is monitored by a zinc-finger domain that has specific or rare targeting sites in the genome [26]. The field of genome editing is progressing by acquiring the ability to engineer and manipulate applied and basic genomic targets.

### 2.3. Transcriptional Activator-Like Effector Nucleases (TALENs)

**Figure 3:**
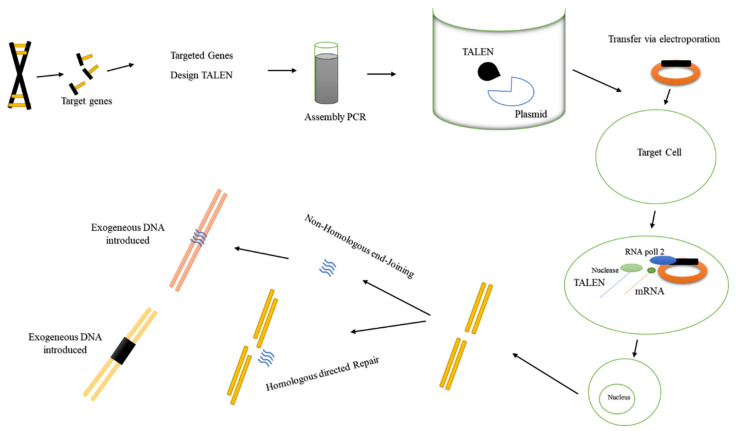
Transcription activator-like effector nucleases (TALENs) are dimeric transcription factors/nucleases engineered from an array of 34-amino-acid molecules, each of which targets one nucleotide. The target sequence is recognized; a corresponding TALEN sequence is built and inserted into a cellular plasmid. The cellular plasmid is inserted into the host cell, where it is translated to produce the functional TALEN, which penetrates the nucleus and binds to and cleaves the target sequence. The applications of this system include the knockout of a target gene or the addition of a replacement nucleotide into the target gene.

The TALEN system for accurate genome editing is a commonly adopted method that has been in use for several years [27]. TALENs were developed via the amalgamation of the *FokI* cleavage domain with the DNA-binding domains of TALE proteins. TALEs comprise multiplex repeats of 34 amino acids for the efficient edition of a single base pair [28]. Like ZFNs, TALENs also promote targeted DSBs that help initiate pathways that are responsible for DNA damage and ensure modifications [4]. The proteins involved in the TALEN system comprise a central domain, which is responsible for binding to DNA, and a nuclear localization sequence [29] (Figure 3). In 2007, it was observed for the first time that these proteins possess the capability of binding to DNA. However, the DNA-binding domain includes a 34-amino-acid repeated sequence, with each repeat perceiving a single nucleotide in the target DNA, whereas each repeated sequence of ZFNs perceived three nucleotides in the target DNA [30].

The number of studies that used ZFNs and TALENs in plants is comparatively small, and these reports appear to favor TALENs; however, the efficiency of editing achieved by these two nucleases is quite low. Therefore, the use of TALENs is more unaffected and favorable for programming. The targets of TALEs are specifically recognized by the occurrence of repeat variable di-residue (RVD) flanking at 12 and 13 positions of each target sequence [26,27].

Generally, TALE proteins can be modulated by binding DNA repeated sequences. Previous studies showed that the nucleotides of the DNA sequence are fixed by the help of TALE proteins always at the 5′ end thymidine base. In the absence of a 5′T, the activities of TALE transcription factors (TALE-TFs) and TALE recombinase (TALE-R) are decreased [31]. TALENs are preferred over ZFNs because their modulation is much simpler and cost effective, with a much lower off-target rate (Table 1).

### 2.4. CRISPR/Cas9

This genome-editing technique, which relies on the activity of RNA-guided nucleases and their mode of action, has gained much attention because of its versatility, potency, adequacy, and simplicity [32]. The CRISPR/Cas9 system is a highly conserved system that originated from the bacterial species *Streptococcus pyogenes* [33,34]. Its discovery was a significant breakthrough of the 20th century, as it represented an entirely distinct and divergent tool that was quickly examined by many bioinformaticians, biotechnologists, and microbiologists.

**Figure 4:**
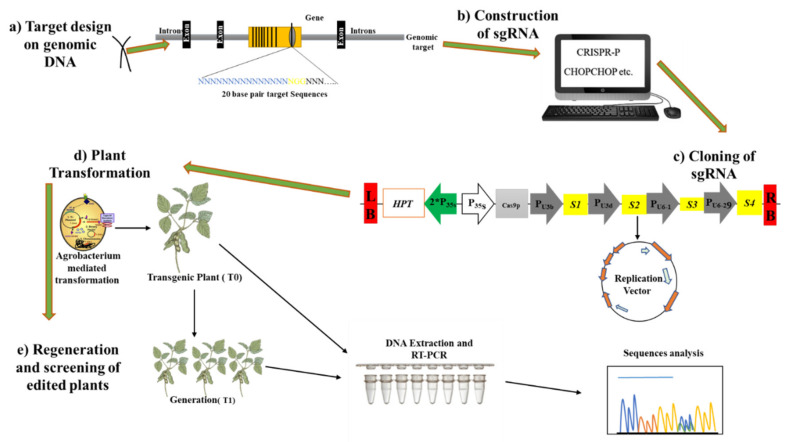
CRISPR/Cas9-based genome editing. (**a**) Selection of the desired target on genomic DNA and recognition of protospacer adjacent motif (PAM) sequences before 20-base-pair sequences. (**b**) Design of the sgRNA using different online bioinformatics tools. (**c**) Subsequent step toward the cloning of designed sgRNAs and the construction of the binary vector using different promoters. (**d**) Transfer of the vector into the plant by *Agrobacterium tumefaciens-*mediated plant transformation and development of transgenic plants. (**e**) Genotyping analysis of transgenic plants, as mentioned in the figure.

In the 2012–2013 period, the CRISPR/Cas9 system was successfully implemented with remarkable cutting efficiency and simplicity to modify animal and plant genes [35,36]. Studies reported three CRISPR/Cas systems (I, II, and III), each of which has distinct molecular mechanisms for nucleic acid piercing and targeting [33,37]. The initial identification of Cas9 (formerly known as COG3513, Csx12, Cas5, or Csn1) through bioinformatics analyses revealed that it acts as a large multifunctional protein structure that comprises two nuclease domains, HNH and RuvC-like [38]. The development of the CRISPR system proved to be advantageous for the manipulation of genetically modified cells in living organisms, as well as in culture (Figure 4) [39].

Because of its versatility, simplicity, efficacy, and wide range of applications, the CRISPR/Cas9 system has been applied in many fields of research, such as biotechnology, genetic engineering, and fundamental and applied biology (Table 1).

With the expansion of the plant genome-editing system, the expression cassette of CRISPR/Cas9 is transformed into the cells, incorporated into the nuclear genome, and expressed, followed by the cleavage of its target DNA sequence, usually 3 bp upstream of the protospacer adjacent motif (PAM) site. Double-stranded breakage of DNA activates two separate mechanisms of DNA repair, NHEJ and homology-directed repair (HDR) [40]. In the absence of a homologous template, NHEJ mediates the direct re-ligation of the broken DNA molecules, normally leading to insertions and deletions (InDels), or substitutions at the DSB site. However, in the presence of a donor DNA sequence, HDR may add new alleles, correct existing changes, or insert new sequences of interest [15,41]. Although DNA becomes integrated into the plant genomic site at a low frequency [42], the integrated transgene can still be expressed and becomes functional only for a short period. Therefore, the expression of CRISPR/Cas9 via transgenesis may offer an alternative method for genome editing in plants. Interestingly, two simple and effective methods adopted for genome editing rely on the expression profile of the CRISPR/Cas9 DNA or RNA [43]. For these methodologies, antibiotic and herbicide selection steps are adopted during post-transformation tissue culture and obstructed, which yield plants that regenerate from the induction cells of the callus that functionally express the CRISPR/Cas9 system.

### 2.5. New Tools for Plant Genome Editing

Based on the revolution of molecular biology and the discovery of sequences in the microbial immune system, biotechnologists are now able to manipulate the genome of organisms of interest in a specific and precise way with the aid of CRISPR and its associated Cas proteins. This remarkable genome-editing system is categorized into two broad classes and six subtypes. CRISPR class II has a type V effector termed Cpf1, which can be designed using highly specific CRISPR RNA to cleave the corresponding DNA sequences [44,45]. Cpf1 has various distinct features, such as the ability to target T-rich motifs, the absence of a requirement for trans-activating crRNA, the versatile capacity to induce a staggered double-strand break, and the potential for both RNA processing and DNA nuclease activity; hence, it represents an alternative to Cas9 [46].

The Cpf1 nuclease or Cas12a was recently discovered in *Prevotella* and *Francisella1* at the MIT and the Broad Institute (USA) by Zhang and his team [47]. Regarding its structural configuration, cpf1 belongs to type V among the CRISPR systems and is a monomeric protein comprising 1200–1500 amino acids. It recognizes a 5′-TTTN-3′ or 5′-TTTV-3′ sequence (V = A, C, or G), in some cases as PAM in a DNA sequence, and the whole array consists of nine spacer sequences, which are disassociated with 36-nucleotide-long repeated sequences (Table 1) [48], ultimately leading to a spacer derived from a part of the crRNA that is complementary to the target DNA [49]. One of the unique features that render Cpf1 a highly useful nuclease is the formation of staggered ends. It contains five 5–8-nucleotide-long overhangs depending on the crRNA length at the site of cleavage [50]. These overhangs enable genome manipulation and provide a flexible approach for base editing and epigenetic modulation [51].

**Figure 5:**
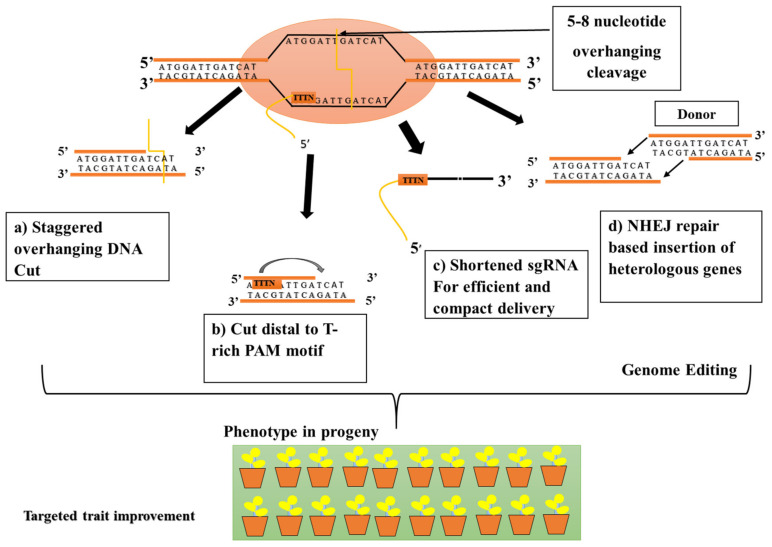
In a Cpf1-mediated plant genome-editing system, the T-rich region (TTTN) acts as a protospacer adjacent motif (PAM). (**a**,**b**) Cpf1 cleaves the target DNA and introduces double-stranded breaks (DSBs), a 5-nt potential staggered cut distal to a 5′ T-rich PAM. (**c**,**d**) In Cpf1, the DSBs are subsequently repaired by two primary cellular mechanisms, nonhomologous end joining (NHEJ) and homology-directed repair (HDR).

Recent reports suggest that Cpf1 can cleave double-stranded DNA at a single catalytic site in the RuvC domain, whereas the Nuc domain is responsible for the regulation of the substrate DNA [52] (Figure 5). Another report suggests that small molecular compounds can enhance the efficiency of Cpf1, as they are directly involved in activating or suppressing signaling pathways for cellular repair. Thus, small-molecule-mediated DNA repair aids CRISPR-mediated knockout strategies [53].

Furthermore, many desirable traits can only be obtained in crops by correctly inserting or removing segments of DNA. Base editing provides a new method for base substitution. However, the conversions of C–T and A–G remain limited [53,54]. Recently, a groundbreaking genome editor, “prime editing,” was developed that can directly insert new genetic information into a designated DNA site, thus dramatically expanding the genome edition range and capabilities [55]. Cas9 is a nickase fused with reverse transcriptase in the prime editing system, and sgRNA is replaced by the prime editing guide RNA (pegRNA), which includes the target site identification of sgRNA and the RNA template specifying the DNA sequence for insertion on the target genome [55].

### 2.6. Applications

#### 2.6.1. MNs, ZFNs, and TALENs

The use of ZFNs was primarily examined in *Arabidopsis* [19,72] and tobacco as model plants. Because of the highly specific nature of the engineered nucleases used for the targeting of genes, researchers believed that this technique with further modifications could be applied to other crop plants [73]. After a few years, many studies reported the results of ZFN-mediated gene-targeting approaches in many crops, including tobacco [18], maize [74], and model plants (*Arabidopsis*) [75]. The application of ZFNs for gene targeting in various plant species is presented in the following sections (Table 2). The only drawback of ZFNs is their ability to bind to any nucleotide sequence (one zinc finger can bind to three nucleotides in the targeted DNA), as well as their ability of binding to off-target sequences [23,76]. Genome editing via ZFNs was also achieved in soybean by targeting DICER-like (*DCL*) genes. The results showed that mutation is comprehensively effective for the transmission of inheritance due to ZFN-induced mutation. The context-dependent assembly scaffold is an open and rapid method that is used for modulating novel ZFN arrays [77].

In addition, findings from previous studies suggest a method for targeted mutagenesis in the genome of paleopolyploid soybean using ZFN that is competent in targeting single or multiple copies of gene families [78]. It has also been reported that the ZFN protein activated the transcriptional machinery of the b-ketoacyl-ACP Synthase II gene in *Brassica napus* [79]. Interestingly, engineered ZFN-TFs can play a significant role in the modification of agronomic traits, as well as endogenous genes [80]. Previous outcomes demonstrated the importance of artificially engineered TALEs, thus allowing the use of TALE-binding code for DNA targeting sites together with TALE DNA-binding domains (DBDs).

Therefore, DBD can be amalgamated with an effector or catalytic domain-like nuclease, e.g., a nuclease, to achieve a remarkable tool for DNA editing [81,82,83]. TALE-fusion proteins employ the C-terminal region of the central repeat domain, which acts as a linker between TALE DBD and the effector domain. The linker length may vary according to the effector domain that is used for the dimerization of the *FoKI* nuclease domain [84,85,86]. However, the length of the longer linker used for the activation of the domain is 65 amino acids. The presence of the *DELLA* gene in tomato, known as *PROCERA* (*PRO*), yielded an inhibitory effect on the regulation of the signaling cascade of gibberellic acid, whereas TALEN edited the *PRO* gene under the control of an estrogen-inducible promoter, resulting in phenotypes with a consistently increased GA response [87].

#### 2.6.2. CRISPR/Cas9 and CRISPR/Cpf1

CRISPR/Cas9 and CRISPR/Cpf1 bring a revolutionary change into the field of biology, and many laboratories around the world are adopting this leading-edge technology because of its tremendous applications. In this section, we summarize the advantages of this powerful approach to engineer genes and their functions for crop improvement.

##### Improvement in Yield and Quality via CRISPR/Cas9 and CRISPR/Cpf1

Genome-editing technologies have far-reaching large-scale practical applications to overcome one of the key milestones of modern biotechnology, i.e., the development of new crop varieties with high yield, resistance to biotic and abiotic stresses, and high nutritional value. Much progress has been achieved using the CRISPR technology. Recently, an oil known as “biotech oil” was obtained from *Camelina sativa* seeds that has wide applications with an enhanced fatty acid composition. It is not only beneficial for human health because of its potency to resist oxidation but also applicable for the production of chemicals that are synthesized commercially, such as biofuels [97,98]. Genomic editing using CRISPR/Cas9-mediated technology has also been used in woody species, such as poplar *(Populus tomentosa Carr).* The phytoene desaturase 8 (*PtoPDS-8*) gene was edited in a site-specific manner using four sgRNAs. A phenotypic analysis of transgenic poplar plants showed an albino phenotype with about 51% of induced mutation frequency [99]. The 4-coumarate: CoA ligase-1 and 4-coumarate: CoA ligase-2 (*4CL1* and *4CL2*) genes also participate in the synthesis of lignin and flavonoid in poplar plants. Using CRISPR/Cas9, the *4CL1* and *4CL2* gene families were mutated under the control of the ubiquitin-6 (*U6*) promoter of Medicago, resulting in the generation of a bi-allelic mutation with a 100% efficiency. The homologs and multiplex recombination-mediated editing of the arabidopsis phytoene desaturase (*PDS3*) gene were obtained with a measured frequency of 65–100% [100,101].

Interestingly, Zhang et al. (2016) [102] engineered a multiple CRISPR/Cas9 system that has been used for fast editing and observed the presence of six editing *PYL* gene families of ABA receptors with a 13–93% mutation frequency in the T1 generation in a single transformation experiment. The brassinosteroid insensitive 1 (*BRI1*), jasmonate-zim-domain protein 1 (*JAZ1*), and gibberellic acid insensitive genes were also engineered using CRISPR-Cas9, with a mutation frequency of 26–84% [42,69]. The flowering Locus T (*FT*) and squamosa promoter-binding protein-like 4 genes were also edited with CRISPR/Cas9; 90% of plants in the T1 generation carried a mutation in the late flowering stage [100]. Mutagenesis of the green fluorescent protein gene in *Nicotiana benthamiana* using Cas9 RNA-guided endonuclease has been ameliorated [103]. This system was transformed using a tobacco rattle virus vector to modulate the regulation of plant genes via engineering and to edit transcriptional factors [104].

The generation of homozygous rice was edited using the CRISPR/Cas9 system by Zhang (2014) and Zhou (2014) [105,106]. The results of these studies suggest that deletions in the chromosomal gene cluster, as well as small heritable variations in the genetic makeup during the genome editing by CRISPR-Cas9, were present in T0 plants. CRISPR/Cas9-mediated mutagenesis was also examined in three members of the rice aldehyde oxidase (*AOX1*) gene family (*OsAox1a*, *OsAox1b*, and *OsAox1c*) and in the OsBEL protein of rice; moreover, the inherent modification of the transgene in the next generation was also reported by Xu et al. [107]. The barley *HvPM19* gene encodes an ABA-inducible membrane protein that is involved in the upregulation of grain dormancy. Mutation induced by Cas9 in two copies of *HvPM19* yielded a 10% mutation frequency [45,108].

It has been acknowledged that the most extensively used wild-type spCas9 is vigorous in identifying both NGG and NAG PAMs in rice. Other applications for producing high-quality crops using an efficient CRISPR-Cas9 system include seeds with a high concentration of oleic acid oil in *Camelina sativa* and *B. napus,* and the targeting of *ALCATRAZ* genes to enhance pod shattering in *B. napus* [109,110]. CRISPR technology is optimal to generate targeted gene knockouts. However, several essential genes cause seedling lethality when knocked out, and several agronomic traits (such as improved photosynthesis) require gene overexpression [111]. The roles of the grain number (*Gn1a*) and grain size (*GS3*) QTLs were investigated with the help of a CRISPR/Cas9-mediated QTL-editing approach in rice [112].

The CRISPR/Cas9 technique was used mainly as a proof of concept in many vegetable crops, e.g., in cabbage, Chinese kale, and watermelon, to induce mutations in the *PDS* gene [113,114]. In the case of vegetables, CRISPR/Cas9 studies have been performed most frequently in tomatoes, because of the economic value of the crop or the ease of genetic transformation using agrobacterium. Parthenocarpy can be a desirable trait in tomatoes because of consumer preference and treatment purposes [115]. Soyk et al. (2017) [116] indicated that the targeted mutagenesis of the engineered self-pruning 5G (*SP5G*) gene of tomato yielded early flowering and more bush, which in turn resulted in an early harvest. Brooks et al. (2014) [117] magnified the CRISPR/Cas9-mediated obstruction of the ARGONAUTE 7 (*SlAGO7*) gene and observed a needle-like or wiry leaf phenotype in tomato (Table 3). The MADS-box transcription factor-encoding RIPENING INHIBITOR (*RIN*) gene was found to regulate fruit ripening in tomato. This technique was modulated to engineer three target regions within the gene; *RIN* mutant (homozygous) tomato plants displayed incomplete ripening with a low pigmentation (red) rate compared with wild-type plants, which demonstrated the crucial role of RIN in the ripening process [100,118]. Furthermore, orthologs of *GA4* in *B. oleracea*, BolC.GA4.a, were used to induce 10% targeted mutations by Cas9 and led to a dwarf phenotype that was linked with *GA4* knockout [71,100,119,120].

Furthermore, the biosynthesis of steroidal glycoalkaloids (SGAs) in potato was used together with the CRISPR/Cas9 method to target 16α-hydroxylase steroids (*St16DOx*). This research provided two SGA-free potato lines with deletions of *St16DOX* [121]. Similarly, the starch synthase *GBSS* gene has been mutated in potato via CRISPR/Cas9. The mutated lines showed reduced amylose levels and an increased concentration of the amylose/amylopectin ratio [122]. Furthermore, the *SnLazy1* locus, which is the tomato ortholog of *Lazy1*, was edited by CRISPR/Cas9 in *Solanum nigra*, with successful inheritance of the removal of two separate snlazy1-cr alleles and the production of plants with stem development in a relatively downward direction [123].

Genome editing may be the only way to improve this important staple food and fruit. To date, only a small number of fruit-producing species (citrus, tomatoes, watermelons, grapes, or strawberries) with traits inherited from CRISPR/Cas9 via the germline have been recorded [118]. Genome editing of gibberellin biosynthesis has allowed the generation of dwarf fruit trees [124], with the capacity for a high productivity rate through dense planting and decreased usage of water and fertilizers and lower land and labor costs. Moreover, genome editing for the inhibition of ethylene biosynthesis was found to play an essential role in the fruit-ripening process [125]. Moreover, its signaling pathways led to the development of new varieties with an increased shelf life [93]. The findings of previous research consistently presented a novel *Xanthomonas citri*, which expedited the technique of agroinfusion to transfer CRISPR-Cas9 for targeting the *CsPDS* gene into sweet orange leaves [119].

Because of the high efficacy of genome editing, which does not allow the involvement of foreign DNA, it will be easy for the consumer to utilize genome-edited fruits. A useful prediction was made by a researcher [120,121], who recently determined that gene editing in golden apple protoplasts can be accomplished by adopting similar Cas9/sgRNA RNP complexes, as discussed earlier for genome editing in other crops. Another group investigated a wild-type species of tomato called groundcherry (*Physalis pruinosa*) that produces a high yield of large fruits [39]. In addition, the *PPO* mutation in apples can be considered transgene free using CRISPR/Cas9 and could easily be applicable worldwide [122]. CRISPR/Cas9 is committed to the development of seedless fruits through the modification/mutation of genes responsible for seed formation. In tomatoes, parthenocarpy has also been recorded by knockout or mutation of the *SIAGL6* and *SIIAA9* genes by CRISPR/Cas9 (Table 3) [123]. The parthenocarpy production method controlled by CRISPR/Cas9 can be implemented in fruits, such as citrus, custard apple, grapes, kinnow, peach, and watermelon, among which there is a high demand for seedless fruit. CRISPR/Cas9 has also recently been used to cause mutations in the *MaGA20ox2* gene, which regulates banana dwarfism [126].

The CRISPR/Cpf1 method has been used to edit the *FAD2-1B* and *FAD2-1A* genes to enhance the oil composition of soybean to produce high-yielding soybean plants with higher oleic acid levels [127]. Using CRISPR/Cpf1, plant breeders have correctly improved production and quality with a high degree of effectiveness [128]. Various Cpf1 proteins were used to mediate the editing of the genomes among various higher plant species, such as tobacco, soybean, and rice. In recent years, the *OsPDS* and *OsBEL* genes were targeted by Cpf1 and were engineered by selecting two genomes within rice for stability and heritage mutations [124,125]. The Chlorophyllidea oxygenase *(CAO1*) gene, which converts chlorophyll a into chlorophyll b, has been targeted for gene insertion in rice using CRISPR/Cpf1 [67,124]. Gene editing using the guide gRNA-Cas9/Cpf1 ribonucleoprotein (RNP) is suitable for fruit tree protoplasts that have been displayed in apple and grape cells [120].

##### Upgrading of Climate-Resilient Crops, Vegetables, and Fruits

The CRISPR technology is widely used together with a variety of biotic and abiotic stresses in major crop plants, such as wheat, rice, corn, cotton, soybeans, tomato, and potato. The CRISPR tool has modernized plant breeding programs for the production of smart climate abiotic stress-tolerant crops. Moreover, CRISPR/Cas9 is a novel technique that can be used to knock out the eukaryotic translational initiation factor *eIF4E* gene, which is necessary for the translation process in vegetables, such as *Cucumis sativus*. The resultant gene knockout ensures resistance against viruses, such as the papaya ringspot mosaic virus-W (*PRSV-W*), the zucchini yellow mosaic virus (*ZYMV*), and the cucumber vein yellowing virus (*CVYV*) [148].

Rice production is significantly decreased by high levels of salt in the soil. The mechanism of salt tolerance in rice was determined by CRISPR/Cas9. A CRISPR/Cas9-mediated knockout mutant of the *OsPRX2* gene exhibited a higher level of antioxidant induction compared with the usual reactive oxygen species (ROS) accumulation [149]. ROS perform an important role in plants by acting as signaling molecules for gene expression regulation, viral pathogen protection, and the symbiotic fixation of nitrogen between the plant and soil rhizobia [150,151].

The CRISPR-Cas9 advanced breeding technology has enabled the development of a new *ARGOS8* variant in maize. Compared with wild-type alleles, the *ARGOS8* variant showed an improved grain yield under flowering stress conditions (five bushels per acre). These findings demonstrated that the CRISPR/Cas9 system is an accurate tool for the generation of new allelic variations in crops for the growth of drought-resistant plants [152]. Furthermore, the knockout of two genes *Drb2a* and *Drb2b* via CRISPR/Cas9 identified their role in controlling salt and drought tolerance in soybean [153]. In tomatoes, molecules, such as mitogen-activated protein kinases, which are responsible for drought stress under the protection of membrane cells against oxidation and via the regulation of transcription genes to manage dry stress, are significant signals. The control of the drought tolerance mechanism via the *SIMAPK3* gene was reported in the tomato system, which produces knockout mutants of the *SlMAPK3* gene under dry stress using CRISPR/Cas9 (Table 4) [154].

Recent studies reported the editing of vegetable crops using CRISPR/Cas9 for the development of different valuable traits. The CRISPR-Cas9-mediated genome editing for heat tolerance was achieved by targeting the SlAGAMOUS-LIKE 6 *(SIAGL6)* gene in tomato. Knockout of the *SIAGL6* gene improved the fruit setting of tomato under heat stress [123]. Similarly, another group used a different approach to develop knockout mutations in the *SIIAA9* gene, which were involved in the auxin signaling pathway to repress the initiation of the development of fruits without fertilization. In addition, in other horticultural plants, such as watermelon, bitter gourd, amber gourd, etc., seedless fruits or fruits with less seeds can be obtained using this precise and rapid method of developing parthenocarpy [115]. In grapes, knockout of *WRKY52*, which encodes a transcription factor related to biotic stress responses, via CRISPR/Cas9 enhanced resistance to *Botrytis cinerea* [155].

In the presence of 100 mM NaCl, self-pollinated offspring tomato plants, which bear the HKT1;2 HDR allele, exhibited stable inheritance germination tolerance. Transgene-free edited plants that reproduce asexually and sexually have been developed using CRISPR/Cpf1 [156]. Hence, the studies mentioned above revealed that CRISPR/Cas9 plays an important role in the development of climate-resilient crops, vegetables, and fruits.

##### Application of CRISPR/Cas9 and CRISPR/Cpf1 to Plant Disease Resistance

Recent advances have been made that cover the major area of genome-editing applications in plant breeding to generate varieties that are resistant against pathogen attack. The adopted methods have been used for the alteration of plant immunity at several stages in different crops [163]. For example, wheat genotypes showed resistance to powdery mildew via the genome editing of the mildew resistance locus O (*MLO*) gene using the TALEN and CRISPR-Cas9 techniques [91]. Genome-editing technology has also been implicated in the generation of resistant plant lines against the bacterial leaf blight caused by *Xanthomonas oryzae* pv. *oryzae* [10,164]. The CRISPR/Cas9 system has been monitored for its ability to provide resistance against geminivirus infection, and geminivirus resistance has been established in both *Arabidopsis* and *N. benthamiana* by introducing sgRNA/Cas9 [165] (Table 5). Another successful modification was afforded by the promoter of *CsLOB1*, which rendered the resulting homozygous plants resistant to *Citrus canker* [157]. The comparative measured mutation rate was 3.2–3.9%, with no off-target effects. CRISPR-mediated editing in the grape cultivar “Chardonnay” showed that the targeted L-idonate dehydrogenase (*IdnDH*) gene exhibited a 100% mutation frequency rate 

Recently, resistance to bacterial blight in rice has been improved through CRISPR/Cas9 by editing the *SWEET11*, *SWEET13,* and *SWEET14* genes [166]. In tomatoes, downy mildew resistance 6 (*DMR6*) knockout mutants exhibited improved wide-spectrum resistance to multiple pathogens, including bacteria and oomycetes [167]. Similarly, the LATERAL ORGAN BOUNDARIES 1 transcription factor (CsLOB1) stimulates the proliferation of *Xanthomonas citri* ssp, which was reported as a causative agent of *Citrus canker* [168]. Moreover, several other studies have shown that CRISPR/Cas9 is effective in generating resistance against viruses in plants, such as the tomato yellow leaf curl virus (*TYLCV*) and the bean yellow dwarf virus (*BeYDV*) [165,169]. The delivery of sgRNAs targeting Cas9-pressing tobacco, including *TYLCV*, in the intergenic region, coat protein (CP), and the viral accumulation of several important viruses [169]. Furthermore, the ethylene-dependent pathway in rice has been modified and mutated successfully via CRISPR/Cas9 edition of the *OsERF922* gene, with the resulting plants showing improved resistance to *Magnaporthe oryzae* [170].

Similarly, two genes of tobacco, *NtPDS* and *NtPDR6*, which encode pleiotropic drug resistance, were modified and mutated with CRISPR/Cas9, resulting in indel frequencies of 16.2–20.3% in protoplasts. Transgenic plants exhibited mutation rates of 81.8% and 87.5% in *NtPDS* and *NtPDR6*, respectively, whereas no significant effect was found near the off-target sites [104]. Furthermore, knockout of the gene encoding the eukaryotic translation initiation factor isoform 4E (*eIF(iso)4E*) in *Arabidopsis* using CRISPR/Cas9 resulted in the enhancement of turnip mosaic virus resistance but did not affect plant vigor [171]. A similar work reported in 2016 showed that the generation of two mutation sites in the *eIF4E* gene by CRISPR/Cas9 in cucumber resulted in resistance to the cucumber vein yellowing virus (*CVYV*), zucchini yellow mosaic virus (*ZYMV*), and papaya ring spot mosaic virus-W (*PRSV-W*) [172].

Such CRISPR/Cas9 applications collectively indicate that it is an essential tool of genome-editing technology and a key participant in the implementation of plant disease resistance.

## 3. Speed Breeding and MAS Using Genome-Editing Tools

The growing human population and changing environment entail several global concerns related to food security [184]. In the early 1990s, molecular markers were commonly used to select the most appropriate breeding lines [185], followed by genomics-assisted breeding in later years [186]. The CRISPR/Cpf1 tool was utilized for plant genome editing in 2016 [67]. Recently, marker-assisted selection (MAS) emerged as an important tool for genome editing. However, the method of implementation of this technique may vary with the advent of modern technologies [187,188]. A group of researchers presented rice as an example of how different mechanisms can be employed to develop an efficient tool to implement genetic variation for crop improvement [189]. Similarly, progress in maize breeding was made by integrating advances in sequencing, genotyping, and transformation, which includes doubled haploid technology and genome editing [190]. Recently, the “Sorghum QTL Atlas” provided an accessible research platform to deploy gene discovery among several species [191]. Subsequently, this concept was demonstrated in barley [192] and legume crops [193,194]. The availability of sequence information has led to more efficient breeding techniques.

“Speed breeding” (SB) is another magic tool that recently obtained notable attention. SB not only shortens the breeding cycle but also accelerates crop research through rapid generation advancement [195]. This technique utilizes artificial light coupled with temperature conditions to accelerate the crossing and inbreeding of various varieties. According to a recent study, the generation time is significantly reduced by providing a 22-h photoperiod and controlled temperature in several crops, such as spring bread wheat (*Triticum aestivum*), durum wheat (*T. durum*), barley (*Hordeum vulgare*), chickpea (*Cicer arietinum*), pea (*Pisum sativum*), canola (*Brassica napus*), the model grass *Brachypodium distachyon*, and the model legume *Medicago truncatula*, compared with the field or a greenhouse with no supplementary light [184,188,196]. Researchers believe that SB holds great potential via its integration with other modern crop technologies, such as high-throughput genotyping, genome editing, and genomic selection, to speed up the rate of crop improvement. The upcoming decade will witness the use of these powerful genome-editing technologies in combination with SB to enhance the speed and impact of better plant genotypes for farmers and consumers worldwide.

## 4. Speed Editing Strategy for Gene-Family Members

**Figure 6:**
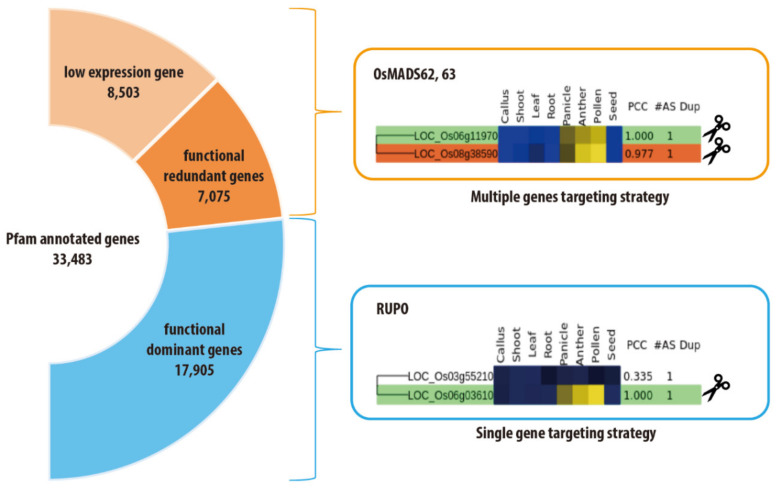
Case studies using the speed editing strategy for gene-family members: Rice contains 33,483 Pfam annotated genes. Among them, the functional significance of homologous genes within a family can be evaluated by integrated transcriptome data and Pearson’s correlation coefficient. *OsMADS62* and *OsMAD63* in the same sisternode of the phylogenetic tree showed redundant expression in mature pollen with the highest expression, and their double mutant exhibited only a male sterile phenotype via a multiple CRISPR/Cas9 system. Conversely, *RUPO* showed predominant expression over *LOC_Os03g55210* in mature pollen, and a single mutation of the *RUPO* gene via the CRISPR/Cas9 system caused a male sterile phenotype.

Recently, we developed a web tool to estimate the functional redundancy of rice genes, because more than 60% of the rice genome has multiple members in the same gene family, as assessed based on Pfam annotation. Functional redundancy associated with gene-family members is one of the main obstacles to crop improvement through gene-editing mediated by a loss-of-function method. Although a gene-editing system involving multiple genes was established in plant species, the editing of multiple genes at a time is generally a complex process. To achieve multiple-gene editing more effectively, we need to select candidate genes with functional redundancy more precisely by considering both protein sequence similarity and coexpression patterns among homologs. Although homologous genes account for more than half of genomes, 7075 out of 33,483 rice genes composing 2617 Pfam gene families retained a Pearson’s correlation coefficient (PCC) value of >0.7 for meta-expression data of anatomical samples, which suggests the existence of other genes with similar function; moreover, 8503 genes exhibited a very low level of expression, which hampers the estimation of their function based on expression data. Therefore, 46.5% of rice genes with gene-family members might not be suitable targets for gene-editing applications using a single target. In addition, the 7075 genes with other family members in the genome having higher similarity in both sequence and expression patterns are more probable targets for multiple-gene editing (Figure 6) [197]. For example, *OsMADS63* genes, which share expression in mature pollen with *OsMADS62*, did not yield a defect in pollen development, whereas multiple mutations of *OsMADS62* and *OsMADS63* with a PCC of 0.977 caused a severe defect in late pollen development, and *RUPO* mutation (PCC, 0.335) over *LOC_Os03g55210* in the same family led to a severe defect in late pollen development and did not require multiple-gene editing. Therefore, accurate estimation of gene redundancy within a family will accelerate crop improvement through gene-editing systems. This web tool (CAFRI-Rice, http://cafri-rice.khu.ac.kr/) is only available for rice, but we expect its expansion to other crop species [197].

## 5. Future Directions

The adopted CRISPR system and its usage will promote the rapid progress of crop breeding and functional genomics. Recently, new and versatile breeding technologies have been implemented to facilitate the engineering of multiple genetic loci in different breeding varieties, which will improve food security and strengthen crop amelioration. Moreover, the perusal of the literature for genomic sequences and their functions is a prerequisite for efficient genome editing. In the future, we will likely witness the increased use of CRISPR for clarifying genomic structures and their role in plants, such as the transcriptional regulation of Cas9 and Cpf1, the monitoring of genetic loci and mechanisms, and the regulation of promoter activity. Moreover, it will also include the modification and identification of epigenetic behavior in communicating the stable relationships between single-nucleotide polymorphisms (SNPs), which are investigated by genetic traits, and genome-wide association studies. Interestingly, this technique was designed to achieve phenotypic characterization in the T_0_ generation by engineering a genome-wide mutant library in rice. Upon consideration of the highly efficient editing achieved in the T_0_ generation, CRISPR/Cas9 was used to engineer a genome-wide mutant library in rapeseed, which will promote gene characterization and its beneficial applications at a later stage.

**Figure 7:**
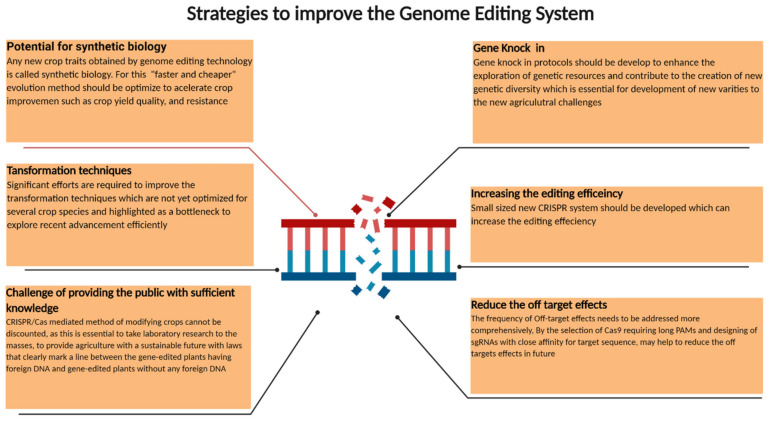
The illustration represents the major strategies aimed at improving the genome-editing systems.

The CRISPR technology can classify any new crop traits in the category of plant synthetic biology (Figure 7). In our opinion, the saturation mutagenesis induced by CRISPR could be used to develop any desired plant protein when a proper selection tool is available. The use of this “faster and cheaper” method of evolution to optimize the role of metabolic enzymes in traits, such as crop production, quality, and disease resistance, should accelerate crop development; however, the CRISPR-associated technology would need to be strengthened. For example, improvements in the transformation methods and delivery of CRISPR/Cas agents to target cells will enable CRISPR in different tissues, including germline cells, and will increase the compatibility of plant species. However, the off-target issue is a big challenge in the application of gene-editing technology and a recent whole-genome sequencing analysis of CRISPR/Cas9-edited cotton plants revealed rare off-target mutations [198]. The detailed strategy to increase on-target and reduce off-target effects of CRISPR/Cas9 was recently well reviewed [199]. Targeted genome editing in rice using chemically modified donor DNA, which are designed for UTR or prompter region and the homology-directed repair method, was successful [200] and further improvements are expected in the future. Creating a large population of CRISPR/Cas9-driven mutagenesis of promoters for developmental genes of tomato contributes to increased genetic variations [201]. It is reasonable to expect that the development of various precision genome-editing technologies for targeted and precise gene/allele replacement, in combination with conventional breeding practices, will expedite the breeding of diverse elite crop varieties for the development of sustainable agriculture.

## 6. Conclusions

Genome editing is becoming the most used and versatile tool for crop improvement and functional genomics. The attractive survival landscapes, such as the efficiency, multiplexing, integrity, and simplicity, as well as the highly specific nature, of the genome-editing technologies mentioned here indicate the manner in which crop breeding is carried out and pave the way for plant breeding for the next generations. This new strategy for crop improvement has proven to be efficacious based on a review of the literature on transcriptomics, biotechnology, genomics, and phonemics. The regulation of transgenic crops was also coherently simplified to support the rapid progression of this technology and render these crops acceptable for consumer usage. In addition to these social and technical challenges, the CRISPR technology was used for the first time to edit plant genomes. Therefore, the use of genome editing on a large scale for crop improvement is already a reality. The journey of genome editing raises ethical questions that need to be addressed by researchers and society on a massive scale.

## Figures and Tables

**Table 1:** Comparison of EMNs, ZFNs, TALENs, CRISPR/Cas9, and CRIPSR/Cpf1.

Functions	EMNs	ZFNs	TALENs	CRIPSRs/Cas9	CRIPSRs/Cpf1	References
**Mode of action**	Information strand directs conversion(s) within the target region	Double-strand breaks in the target DNA	Double-strand breaks in the target DNA	Double-strand breaks or single-strand nicks in the target DNA	Double-strand breaks	[56,57,58,59]
**Target recognition** **efficiency**	High	High	High	High	Very High	[59,60]
**Mutation rate**	Middle	High	Middle	Low	High	[4,56,59]
**Creation of large-scale libraries**	Technically difficult	Impossible	Technically difficult	Possible	Possible	[59,61,62]
**Multiplexing**	Technically difficult	Difficult	Difficult	Possible	Possible	[56,57,59]
**Components**	Exogenous polynucleotide (chimeraplast)	Zn finger domains Nonspecific FokI nuclease domain	TALE DNA-binding domains Nonspecific FokI nuclease domain	crRNA, Cas9 proteins	crRNA, Cpf1 proteins	[59,60,63]
**Structural** **protein**	Dimeric protein	Dimeric protein	Dimeric protein	Monomeric Protein	Monomeric Protein	[4,56,59]
**Catalytic** **Domain**	Absence of a catalytic domain	Restriction endonuclease FokI	Restriction endonuclease FokI	RuvC and HNH	RuvC and HNH	[59,63,64]
**Length of the target sequence (bp)**	68–88	24–36	24–59	20–22	20–24	[4,61,65]
**Protein** **engineering** **steps**	Not required	Required	Required	Should not be difficult to test gRNA	Should not be difficult to test gRNA	[59,62,66]
**Cloning**	Not necessary	Necessary	Necessary	Not necessary	Not necessary	[59,62,66]
**gRNA** **production**	Not required	Not applicable	Not applicable	Easy to produce	Easy to produce	[59,62,67]
**Target genome-editing tools**	Not Required	ZFNGenome v2.0 ZifBASE Zinc-Finger Database (ZiFDB) Zinc-Finger Tool EENdb	TALE-NT 2.0 SPATA TALEN offer TALEN Library T	CHOP CHOP CRISPRs web Server Crass: The CRISPR Assembler CRISPR Target	Breaking-Cas Cas-OFFinder CRISPOR CCTOP	[46,68]
**Off-target** **effects**	Low off-target effect	Low off-target effect	Shows least off-target activities	Low off-target effect	Low off-target effect	[69]
**Cost of development**	High	High	Higher	Low	Low	[63,70,71]

**Table 2:** Improvement in crops, fruits, and vegetables via EMNs, ZFNs, and TALENs.

Tools	Crop/Fruits/Vegetable	Target Gene	Trait Improvement	References
EMNs	Maize	*MS26*	Independent lines of male sterile plants	[88]
EMNs	Cotton	*EPSPS*	Herbicide tolerance	[89]
ZFN	Soybean	*DCL*	Herbicide transmission	[77]
ZFN	Maize	*PAT*	Herbicide resistance	[29]
ZFN	Tobacco	*GUS: NPTII*	Chromosome breaks	[73]
ZFN	RICE	*OsQQR*	Detection of safe harbor loci Herbicide	[90]
TALEN	Wheat	*TaMLO*	Powdery mildew resistance	[91]
TALEN	Potato	*Endogenous consist. Promoter*	Herbicide resistance	[4]
TALEN	Potato	*ALS*	Herbicide resistance	[92]
TALEN	Potato	*Vacuolar invertase*	No reducing sugars and improved food safety	[93]
TALEN	Sugarcane	*Caffeic acid O-methyltransferase*	Reduced lignin and improved biofuel production	[94]
TALEN	Potato	*Vlnv*	Low concentration of reducing sugars and undetectable concentration of reducing sugars	[93]
TALEN	Rice	*OsBADH2*	Fragrant rice	[95]
TALEN	Soybean	*FAD2-1A, FAD2-1B*	Low polyunsaturated fats	[96]
TALEN	Wheat	*TaMLO-A1, TaMLO-B1, TaMLO-D1*	Powdery mildew resistance	[91]

**Table 3:** Improvement in the yield and quality of crops, fruits, and vegetables via CRISPR.

Tools	Crop/Fruit/Vegetable	Target Gene	Trait Improvement	References
CRISPR/Cas9	Rice	*Gn1a, GS3, and DEP1*	Grain number, grain size, panicle architecture	[129,130]
CRISPR/Cas9	Wheat	*TaGASR7*	Grain length and weight	[131]
CRISPR/Cas9	Flax	*FAD2*	Seed oil composition (high oleic and low polyunsaturated FAs)	[97]
CRISPR/Cas9	Soybean	*GmFT2a*	Late flowering	[132]
CRISPR/Cas9	Tomato	*SP5G*	Time to harvest	[116]
CRISPR/Cas9	Tomato	*RIN*	Fruit ripening (shelf life)	[133]
CRISPR/Cas9	Tomato	*SlIAA9*	Parthenocarpy (leading to seedless fruit)	[115]
CRISPR/Cas9	Wheat	*PDS*	Chlorophyll syn	[57]
CRISPR/Cas9	Cotton	*ALARP*	Cotton fiber development	[134]
CRISPR/Cas9	Rice	*Waxy*	Enhanced glutinosity	[135]
CRISPR/Cas9	Rice	*Hd2, Hd4, Hd5*	Early heading	[136]
CRISPR/Cas9	Maize	*PPR, RPL*	Reduced zein protein	[137]
CRISPR/Cas9	Potato	*GBSS*	Increased amylopectin/amylose	[138]
CRISPR/Cas9	Sorghum	*Wholek1Cgene family*	Increase in the grain protein digestibility and lysine content	[139]
CRISPR/Cas9	Petunia	*PDS*	The biosynthesis of carotenoid and chlorophyll	[140]
CRISPR/Cas9	Carrot	*DcPDS, DcMYB113*	Purple depigmented carrot	[141]
CRISPR/Cas 9	Cabbage	*Bolc.GA4.a*	Dwarfing and fruit dehiscence	[108]
CRISPR/Cas9	Grape	*VvPDS, MLO-7*	Albino phenotype	[142]
CRISPR/Cas 9	Banana	*PDS*	Albino and variegated phenotype	[143]
CRISPR/Cas 9	Watermelon	*ClPDS*	Albino phenotype	[144]
CRISPR/Cas9	Apple	*PDS, TFL1*	Albino phenotype, early flowering	[145]
CRISPR/Cpf1	Tobacco	*ETR1*	Plants harboring	[146]
CRISPR/Cpf1	Maize	*PAP1*	Stable mRNA equal	[47]
CRISPR/Cpf1	Rice	*OsROC5, OsDEP1*	Mutation frequencies doubled	[47]
CRISPR/Cpf1	Rice	*OsEPFL9*	Regulation of stomatal density	[147]

**Table 4:** Improvement of climate-resilient crops, vegetables, and fruits by CRISPR/Cas9/Cpf1.

Tools	Crop/Fruit/Vegetable	Target Gene	Trait Improvement	References
CRISPR/Cas9	Maize	*ARGOS8*	Drought tolerance	[157]
CRISPR/Cas9	Rice	*OsNAC041*	Salinity tolerance	[158]
CRISPR/Cas9	Tomato	*NPRI*	Drought tolerance	[159]
CRISPR/Cas9	Soybean	*Drb2a and Drb2b*	Salt and drought tolerance	[153]
CRISPR/Cas9	Tomato	*SIMAPK3*	Drought tolerance	[154]
CRISPR/Cas9	Tomato	*SIAGL6*	Heat stress	[123]
CRISPR/Cas9	Grapes	*WRKY52,*	Biotic stress responses	[155]
CRISPR/Cas9	Soybean	*SAPK1 and SAPK2*	Salinity tolerance	[160]
CRISPR/Cas9	Maize	*ZmHKT1*	Salinity tolerance	[161]
CRISPR/Cas9	Rice	*OsMPK2, OsPDS, OsBADH2*	Multiple-stress tolerance	[162]
CRISPR/Cpf1	Tomato	*HKT1;2 HDR*	Multiple-stress tolerance	[156]

**Table 5:** Improvement of plant disease resistance by CRISPR in crops, fruits, and vegetables.

Tools	Crop/Fruit/Vegetable	Target Gene	Trait Improvement	References
CRISPR/Cas 9	Citrus (orange)	*CsLOB1 (promoter)*	Citrus canker resistance	[157]
CRISPR/Cas 9	Cucumber	*eIF4E*	Broad virus resistance	[172]
CRISPR/Cas 9	Tobacco	*43 regions in the viral genome*	Resistance to the Gemini virus beet severe curly top virus	[173]
CRISPR/Cas 9	Tobacco	*Six regions in the viral genome*	Resistance to the Gemini virus bean yellow dwarf virus	[174]
CRISPR/Cas 9	Tomato	*Three regions in the viral genome*	Resistance to the Gemini virus Resistance to the tomato yellow leaf curl virus	[175]
CRISPR/Cas 9	Tomato	*SlMlo1*	Resistance to powdery mildew	[176]
CRISPR/Cas 9	Wheat	*MLO-A1, TaMLO-B1 and TaMLO-D1*	Resistance to powdery mildew	[91]
CRISPR/Cas9	Grape	*VvPDS, MLO-7*	Albino phenotype Powdery mildew resistance	[120,142]
CRISPR/Cas9	Wheat	*TaMLO*	Powdery mildew resistance	[91]
CRISPR/Cas9	Potato	*S-genes*	*Phytophthora infestans* resistance	[138]
CRISPR/Cas9	Cotton	*Viral and satellite DNAs*	Resistance to cotton leaf curl disease	[177]
CRISPR/Cas9	Citrus	*CsLOB1*	Canker resistance	[178]
CRISPR/Cas9	Apple	*DIPM-1, DIPM-2, and DIPM-4 genes*	Resistance to fire blight disease	[120]
CRISPR/Cas9	Potato	*S-genes*	*Phytophthora infestans* resistance	[138]
CRISPR/Cas9	Rapeseed	*WRKY70, WRKY11*	JA- and SA-induced resistance to pathogens	[179]
CRISPR/Cas9	Rice	*Pi-ta*	Resistance to the rice blast disease	[180]
CRISPR/Cas9	Wheat	*EDR1*	Improved resistance against powdery mildew	[181]
CRISPR/Cas9	Tomato	*SlJAZ2*	Bacterial speck resistance	[182]
CRISPR/Cas9	Cotton	Gh14-3-3	Resistance to cotton verticillium wilt	[183]

## Abbreviations

ZFN	Zinc-Finger Nucleases
TALENs	Transcriptional activator-like Effector Nucleases
CRISPR	Clustered Regularly Interspaced Short Palindromic Repeats (CRISPR)
Cas9	CRISPR-associated Proteins
Cpf1	CRISPR-associated endonuclease in Prevotella and Francisella
DSB	Double-Strand Breaks
NHEJ	Nonhomologous end jointing
HDR	Homology-directed repair mechanism
GMO	Genetically Modified Organism
SB	Speed breeding
RVD	Repeat variable di-residue
